# Placing Salt/Soy Sauce at Dining Tables and Out-Of-Home Behavior Are Related to Urinary Sodium Excretion in Japanese Secondary School Students

**DOI:** 10.3390/nu9121290

**Published:** 2017-11-28

**Authors:** Masayuki Okuda, Keiko Asakura, Satoshi Sasaki

**Affiliations:** 1 Department of Public Health, Graduate School of Sciences for Innovation, Yamaguchi University, 1-1-1 Minami-Kogushi, Ube 755-8505, Japan; 2 Department of Environmental and Occupational Health, School of Medicine, Toho University, 5-21-16 Omori-Nishi, Ota-ku, Tokyo 143-8540, Japan; jzf01334@nifty.ne.jp; 3 Department of Social and Preventive Epidemiology, School of Public Health, The University of Tokyo, 7-3-1 Hongo, Bunkyo-ku, Tokyo 113-0033, Japan; stssasak@m.u-tokyo.ac.jp

**Keywords:** convenience store, restaurants, secondary school students, sodium excretion, salt/soy sauce placed at dining tables

## Abstract

We investigated whether home environment, salt knowledge, and salt-use behavior were associated with urinary sodium (Na) excretion in Japanese secondary school students. Students (267; mean age, 14.2 years) from Suo-Oshima, Japan, collected three overnight urine samples and completed a salt environment/knowledge/behavior questionnaire. A subset of students (*n* = 66) collected, on non-consecutive days, two 24 h urine samples, and this subset was used to derive a formula for estimating 24 h Na excretion. Generalized linear models were used to examine the association between salt environment/knowledge/behavior and Na excretions. Students that had salt or soy sauce placed on the dining table during meals excreted more Na than those that did not (*p_for trend_* < 0.05). A number of foods to which the students added seasonings were positively associated with Na excretion (*p_for trend_* = 0.005). The students who frequently bought foods at convenience stores or visited restaurants excreted more Na in urine than those who seldom bought foods (*p_for trend_* < 0.05). Knowledge about salt or discretionary seasoning use was not significantly associated with Na excretion. The associations found in this study indicate that home environment and salt-use behavior may be a target for a public health intervention to reduce salt intake of secondary school students.

## 1. Introduction

Reduction of salt intake is a challenge worldwide to control health risks such as hypertension, obesity, osteoporosis, asthma, and gastric cancer [1]. The Japanese population is one of the largest consumers of salt (186–231 mmol/day) worldwide [2]. In our previous study, salt intake of Japanese adolescents was estimated to be 10.0–10.6 g/day, which is similar to that of adults [3].

Salt-use behaviors could be a target for interventions to reduce salt intake. In studies of Japanese adults, while knowledge about salt were not associated with 24 h sodium (Na) excretion, behavior such as salty food intake was associated with measured and estimated 24 h Na excretion [4,5]. The association between Na intake and salt-use behaviors, observed in Japanese adults, is unknown in children and adolescents. Salt intake of primary school children was correlated with that of their parents [6,7]; having breakfast and dinner at home likely results in such a correlation. Encouragement to parents may be an intervention measure to reduce salt intake of students. While secondary school children may have dietary habits similar to those they live with, it is likely that they sometimes go out without supervision and independently buy and cook their daily food. Their behavior when selecting and purchasing foods, in addition to their home environment, may affect their salt intake and constitutes a consideration with respect to intervention.

In this study, we investigated whether home environment, salt knowledge, and salt-use behaviors were associated with estimated urinary Na excretion in Japanese secondary school students. These factors, which have the potential to influence salty preferences and habits persisting in adulthood, may be possible targets for public health intervention.

## 2. Materials and Methods

### 2.1. Subjects

The subjects were recruited from 4 secondary schools in the town of Suo-Oshima, Yamaguchi prefecture, Japan, in 2013. There were 320 secondary-school-aged adolescents registered as residents in the town, 300 attended these four schools, and 281 students participated in this study. Of these, 278 subjects, as well as their guardians, gave informed consent. Eleven were excluded from the analysis because 7 subjects did not complete either urine sampling or the questionnaire and 4 had missing values in the questionnaire responses; we therefore analyzed 267 subjects with estimated Na excretion data and its subpopulation 66 subjects with measured 24 h Na excretion data. 

### 2.2. Questionnaire

The questionnaire was distributed at school 1–2 weeks before urine sampling, and 97% of the students filled it out themselves at home, and 3% with their parents’ help. Nine questions on salt intake were about home environment (2 questions), salt knowledge (3 questions), and salt-use behaviors (4 questions; Table 1). Four to six response options for knowledge questions were classified into two categories. Thirteen food items were listed under the question ‘do you add seasoning to the following foods?’ and were classified into five categories (≤2, 3, 4, 5, and ≥6 foods). Foods were selected based on a discussion by the authors about foods for which seasonings were frequently added at the dining table in Japan. The frequency of visiting convenience stores was classified into five categories; options such as ‘more than 3 times a week’ and ‘2 times a week’ were grouped together to make a group size >10 to increase statistical power. Frequency of eating in restaurants was classified into five categories and ‘less than once in six months’ and ‘did not eat’ were grouped together. The questionnaire for the parents was distributed and returned in a closed envelope through their students. The parents were asked about their educational status.

The students reported the average amount of vigorous or moderate activity (≥3 metabolic equivalents) per week in hours and minutes, and they reported their commute time to school (walking or riding a bicycle) in minutes [8]. We calculated the physical activity of the commute per week as one-way × 2 (to and from) × 5 (days/week). Total time of physical activity (min/week) was the sum of vigorous activity, moderate activity, and commuting.

### 2.3. Urine Sampling and Urinalysis

Sampling and analysis methods have been described in detail previously [3]. Briefly, three non-consecutive overnight urine samples were taken with at least 3 days between each collection between November 2013 and February 2014. Oral and written instructions with urine sampling tools were provided to the students before each sampling. The students were asked to record voiding time and to store 7–8 mL of the overnight specimen (a first void after wake-up in the morning) in a plastic test tube in a cold place at home. The overnight specimen was brought to school, and we collected and refrigerated them in a test tube before analysis. Out of all students, 66 collected 24 h urine samples twice to measure 24 h Na excretion (Supplementary Information). All frozen samples were transferred and analyzed using the LSI Medience Corporation (Tokyo, Japan). Sodium (Na) and potassium (K) concentrations (mmol/L) were analyzed using the electrode method. The amount of 24 h sodium (24 h Na; mmol/day) excretion was estimated from overnight as follows:

Estimated Na (mmol/day) = 131.713 + 0.283536 × Na concentration of overnight urine (mmol/L) − 0.861 × K concentration of overnight urine (mmol/L) + 0.029 × total time of physical activity (min/week)(1)

Estimated 24 h Na excretion was derived using Equation (1) [3] for each overnight urine collection. An average of the three estimates from each person was used for analysis.

### 2.4. Demographic, Anthropometric, and Socioeconomic Variables

Chronological age was defined as the difference between birth date and the date of the first urine sampling. We inquired about the education of the students’ parents, considering that higher educational status of the parents is associated with the student’s socioeconomic status, and divided it into three categories. “Low” was defined as completing secondary or high school, “middle” as completing junior college or vocational school, and “high” as completing college or graduate school. Two weeks before the first urine sample was taken, the subjects’ body height and weight were measured by school nurses while the subjects were clothed only in their underwear. Height (cm) and weight (kg) was measured to one decimal place, and body mass index (BMI) was calculated as weight (kg)/height (m)^2^. 

### 2.5. Statistical Analysis

Variables are described as mean and standard deviation (SD) or frequency and percentage. Differences between boys and girls were examined using a chi-square test or a *t*-test. Generalized linear models were used to examine the association between estimated Na excretion as a dependent variable and salt-related factors such as home environment, salt knowledge, or salt-use behaviors. Each association was tested as a linear trend of estimated Na excretion along levels of a factor. Possible confounders, such as grade, sex, and parent’s education, were included in the models. In addition, BMI and physical activity (min/week) were included. We also used measured Na excretion in a subpopulation as a dependent variable with all cofounders included (Supplementary Information), because physical activity was a predicted variable to estimate Na excretion, and a 24 h urinary collection is the gold standard in measuring daily Na excretion. Interaction terms between salt-related factors and sex were examined, and we analyzed the data stratified by sex (Supplementary Information). Statistical analysis was conducted using SAS 9.4 (SAS Institute Japan, Tokyo), and *p* < 0.05 was considered as significant. 

## 3. Results

The ranges of ages in both sexes were 12.7–15.8 years. Body mass indices of boys and girls were 20.1 (SD, 3.6) kg/m^2^ and 20.6 (SD, 2.9) kg/m^2^, respectively (*p* = 0.263; Table 2). Boys engaged in more physical activity compared to girls (*p* < 0.001). Estimated Na excretion was higher in boys than in girls (*p* = 0.002), but 24 h Na excretion was similar among boys and girls in a subpopulation (153.8 (SD, 40.1) and 157.4 (SD, 49.2), respectively; *p* = 0.762).

Home environment was associated with estimated urinary Na excretion (Table 3). The students that had salt or soy sauce often placed on the dining table at meals excreted more Na than those that did not (*p_for trend_* = 0.001 and 0.004, respectively). Among student behaviors, many foods to which students added seasoning were positively associated with Na excretion (*p_for trend_* = 0.005). The students who frequently bought food and drinks from convenience stores or visited restaurants excreted more Na in urine than those who seldom bought foods (*p* = 0.002, and 0.001, respectively). Differences between the highest and lowest Na-excreted levels, excluding those of the students between ≤2 and ≥6 foods added seasoning (9.3 mmol/day), were >10 mmol/day. These associations did not change after adjusting for grade, sex, and parent’s education (*p_for trend_* = 0.002–0.037). Adding BMI as a confounding factor in the model did not change the associations, but adding physical activity attenuated the associations and rendered them no longer significant. When measured Na excretion, which was calculated independent of physical activity, was used as a dependent variable, the students frequently visiting restaurants excreted more Na in urine even after adjustment for physical activity and BMI (195.0, and 125.0 mmol/day in the highest and the lowest, respectively; *p_for trend_* = 0.03; Table S1). Knowledge about salt or discretional seasoning use was not associated with Na excretion. When interaction terms were included in the models, only the interaction between discretional seasoning use and sex was significant, and discretional use was significantly associated with estimated Na excretion in girls, but not in boys (Tables S2–S4). Other associations in both sexes, most of which were non-significant, revealed trends similar to that from all data.

## 4. Discussion

Estimated daily Na excretion was associated with having salt/soy sauce placed on the dining table during meals and visiting stores/restaurants among secondary school students in this study. Among these associated factors, visiting restaurants demonstrated an association with measured daily Na excretion in addition to estimated Na excretion. 

Few reports mentioned the relationship between restaurants and salt intake. A study based on the National Health and Nutritional Survey reports that adolescents in the United States (US) aged between 12 and 19 years consume 25.9% of dietary salt from restaurants [9]. Visiting restaurants, especially quick-service restaurants, contributed to increased salt intake in the US. In addition, foods consumed while dining out contributed to 27–31% of daily salt intake in Japanese adults, and this proportion was higher in young adults (21–36 years) than in older adults (53–69 years) [10]. A relatively large contribution of out-of-home foods to salt intake in children may increase this association, as seen in previous results. Salt reduction in foods provided out of home may be an intervention measure to reduce population salt intake not only for adults, but also for adolescents.

The circumstances surrounding secondary school students may be different from those surrounding adults. If Japanese secondary school students visit restaurants, they are most likely to visit fast food restaurants with their friends or be accompanied by their guardians, and the restaurants may be in family-friendly locations. In Suo-Oshima, there are no hamburger or pizza takeout/delivery restaurants, but there are a few noodle restaurants that offer, for example, ramen and udon, which contain high salt content. Although the students could visit restaurants outside the town, we were not aware of the types of restaurants they visited. Therefore, information about the stores they visit will aid in formulating interventions to reduce salt intake in children.

The availability of convenience stores has been associated with obesity in adolescents in the US [11,12]. A majority of foods acting as energy sources are obtained from convenience stores (63.3–70.3%) in the US population [13], and frequent utilization of convenience stores influences their nutritional intakes [14]; however, the extent of influence of salt intake is unknown. With regard to salt intake, 58.7% of Na intake among adolescents in the US (12–19 years) was from stores, and processed foods are a major source of salt intake both in Japan and US or Europe [10,15]. Fourteen stores in Suo-Oshima provide food (supermarkets, grocery stores, and convenience stores) and are accessible to secondary school students. The association between nutrient intake and food environment may vary according to countries and areas [16]. A geographical information system could be used to elucidate the association between accessibility to each store and salt intake.

A main source of salt intake in the Japanese population is soy sauce, which is placed on dining tables and used in cooking [15]. In this study, those that never had soy sauce placed at the dining table were less than those that never had salt so placed (25% of the students vs. 52%). Self-reported usage of soy sauce at the dining table has been found to relate to Na excretion in urine in Japanese adults [4]. The results of this study also show associations regarding seasoning placed at the dining table in all populations as well as associations regarding discretionary seasoning use in girls. It may be wise, for salt intake reduction, to keep seasoning, especially soy sauce, away from the dining table.

Knowledge and belief are influential factors before lifestyle change [17]. Previous reports on adults did not show the relationship between salt-related knowledge or belief and salt intake [5,18,19]. In a cross-sectional study, prior knowledge could bias the causal association between knowledge and behavior; however, it is unlikely that the students in this study were aware of their own salt intake or that they tried to improve their dietary behavior. On the other hand, most students of this study (80%; Table 3) had already known that salt intake affected their health. Parents’ salt intake was associated with that of their children [7]. Thus, the home environment of secondary school students, rather than knowledge, influences salt intake, and interventions should be conducted to engage and support parents instead of providing health education to children about the effects of salt on health. 

Our study had some limitations. First, Na intake was estimated using students’ overnight urine samples instead of 24 h urine samples and so is vulnerable to bias. We obtained an estimation formula from the subpopulation sample of this study, and Pearson’s correlation coefficients were 0.312–0.478 between estimated and measured Na excretion [3]. Estimated Na excretion was higher in boys than in girls, while measured Na excretion was similar. This may be because physical activity was used a predictor to estimate Na excretion from overnight urine, and boys engaged in more physical activity than girls. The equation used for estimating Na excretion in this study cannot eliminate influences of physical activity on estimated excretion, which might interfere with evaluations of actual salt-related factor effects and lead to estimated Na excretion levels that are different in boys and girls. However, measured Na excretion calculated without a physical activity variable was related to visiting restaurants, and, even from sex-stratified data, we obtained associations similar to those from all data. Second, the questionnaires were not validated, and self-reported data may be also vulnerable to bias. The students likely provided answers believed to indicate preferable knowledge and behavior, so the associations of the results may be attenuated. Third, Japanese populations have one of the highest dietary salt consumption worldwide [2], which indicates that the results are not applicable to other countries. Furthermore, the subjects were from a small local area and may not even be representative of Japanese students. Cultural, geographical, or social factors may influence dietary habits related to salt intake. In addition, a small sample size might further blur the association; significant associations seen in girls were not demonstrated in boys, and estimated Na excretion levels between those who used seasoning placed at the dining table “sometimes” and those who used such seasoning “rarely” were similar, while overall associations were significant. A study with a large and nationwide sample is necessary before nationwide interventions based on these findings can be implemented.

## 5. Conclusions

We showed an association between home environment and salt-use behavior with respect to Na excretion in Japanese secondary school students. Availability of salt or soy sauce placed on dining tables during meals and visiting stores/restaurants away from home was found to correlate with high Na excretion. These environments and behaviors in children and adolescents may be changeable if their families and the stores/restaurants at which they frequent become subjects of intervention. Educating children and adolescents about salt intake is not likely to help them make the relevant reductions. The results of this study indicate that, for the purpose of reducing salt intake in secondary school students, home environments and out-of-home behavior, in addition to an overall reduction of salt in ready-cooked foods, may be viable targets for public health intervention. We should develop approaches that attempt to influence the students’ home environment and, with the involvement of their parents, the rules they are subjected to at home.

## Figures and Tables

**Table 1:** Questions about home environment, knowledge, and behaviors.

Category	Questions	Options
Home environment	How often is salt placed on the dining table for discretionary use at meals?	1 often 2 sometimes 3 seldom 4 never
	How often is sauce (including soy sauce) placed on the dining table for discretionary use at meals?	1 often 2 sometimes 3 seldom 4 never
Knowledge	Salt intake affects your health.	‘Agree’ 1 agree 2 slightly agree ‘Disagree’ 3 slightly disagree 4 disagree 5 do not know
	What is your salt intake, nutritionally speaking?	‘High’ 4 slightly high 5 extremely high ‘Not high’ 1 extremely low 2 slightly low 3 appropriate 6 do not know
	The salt intake of Japanese adults, compared to adults from other countries, is generally __________.	‘Higher than others’ 3 higher than ‘Not higher’ 1 lower than 2 as much as 4 do not know
Behavior	How often do you add seasoning to seasoned food?	1 often 2 sometimes 3 seldom 4 never
	Which of the following foods do you add seasoning to?	curry and rice, fried egg, raw fish, salted salmon, shredded cabbage, jiao-zi (dumpling), tempura (fritter), natto (fermented boiled soybeans), pickles of Chinese leaves, dried whitebait, boiled spinach, pickled wakame (seaweed), and tofu (bean curd)
	How many times did you buy meals or snacks from a convenience store or visit restaurants within the last year?	Combined 1, and 2 1 more than 3 times in a week 2 twice in a week 3 once in a week 4 between once in a week and more than once in a month 5 less than once in a month 6 did not buy
	How many times did you eat out per week within the last year?	1 more than 2 times in a month 2 twice in a month 3 once in a month 4 between once in a month and more than once in 6 months Combined 5, and 6 5 less than once in 6 months 6 did not eat

**Table 2:** Characteristics of the subjects.

	Boys (*n* = 128)		Girls (*n* = 139)		*p*
Grade ^1^ 1st	38	(29.7%)	50	(36.0%)	
2nd	44	(34.4%)	42	(30.2%)	0.405
3rd	46	(35.9%)	47	(33.8%)	
Parent education^1^ Low	42	(32.8%)	38	(27.3%)	
Medium	46	(35.9%)	41	(29.5%)	0.083
High	40	(31.3%)	60	(43.2%)	
Age ^2^, years	14.2	(40.8)	14.2	(0.9)	0.996
Body mass index ^2^, kg/m^2^	20.1	(3.6)	20.6	(2.9)	0.263
Time of VMPA ^2^, min/week	885.9	(572.4)	635.4	(439.0)	<0.001
Estimated Na excretion ^2^, mmol/day	161.8	(22.0)	153.3	(21.4)	0.002

VMPA: vigorous and moderate physical activity. ^1^ frequency (%), and compared between sexes using a chi-square test; ^2^ mean (standard deviation), and compared between sexes using a *t*-test.

**Table 3:** Estimated Na excretion and related factors (*n* = 267).

	*n*	%	Na Excretion, mmol/day		*p_for trend_* ^1^		
			Mean	(Standard Deviation)	Model 1	Model 2	Model 3
Home environment							
Salt placed at dining table							
Often	48	18.0	165.4	(39.0)			
Sometimes	23	8.6	159.3	(53.5)	0.001	0.002	0.002
Rarely	56	21.0	160.8	(40.7)			
Never	140	52.4	153.0	(46.7)			
Soy sauce placed at dining table							
Often	95	35.6	162.3	(39.0)			
Sometimes	56	21.0	156.3	(45.3)	0.004	0.025	0.024
Rarely	49	18.4	156.6	(45.0)			
Never	67	25.1	151.9	(55.2)			
Knowledge							
Health effect of salt							
Disagree	53	19.9	161.3	(34.1)	0.150	0.793	0.769
Agree	214	80.2	156.4	(47.0)			
Salt intake appropriate for health							
High	104	61.1	157.9	(45.3)	0.758	0.641	0.660
Not high	163	40.0	157.0	(45.9)			
Japanese intake							
Higher than others	101	37.8	156.4	(45.9)	0.357	0.260	0.275
Not higher	166	62.2	159.0	(45.7)			
Behaviors							
Discretional seasoning use							
Often	21	7.9	159.6	(43.3)			
Sometimes	65	24.3	158.6	(50.7)	0.093	0.343	0.290
Rarely	83	31.1	160.9	(38.8)			
Never	98	36.7	153.2	(48.1)			
Add seasoning on foods							
≤2 foods	60	22.5	152.9	(41.6)			
3	44	16.5	151.5	(15.5)	0.005	0.005	0.005
4	64	24.0	159.7	(44.4)			
5	46	17.2	160.0	(51.1)			
≥6	53	19.9	162.2	(44.2)			
Convenience stores							
≥2/week	24	9.0	165.6	(66.5)			
1/week	45	16.9	159.8	(32.5)	0.002	0.037	0.038
<1/week and ≥1/month	66	24.7	159.8	(50.6)			
<1/month	77	28.8	157.0	(35.9)			
Did not buy	55	20.6	149.5	(57.6)			
Restaurant							
>2/month	34	12.7	163.7	(23.5)			
2/month	55	20.6	163.0	(40.4)	0.001	0.016	0.014
1/month	66	24.7	156.7	(40.5)			
<1/month and ≥1/6 months	70	26.2	154.3	(52.5)			
<1/6 months	42	15.7	151.2	(32.9)			

^1^ Model 1 was a crude model, Model 2 was adjusted for grade, sex, and parent’s education, and Model 3 was adjusted for grade, sex, parent’s education, and body mass index.

## Supplementary Materials

The following are available online at http://www.mdpi.com/2072-6643/9/12/1290/s1. Supplementary Information: Measured 24 h Na excretion: Table S1: Measured Na excretion (*n* = 66), Table S2: Effect of interaction terms with sex on estimated Na excretion (*n* = 267), Table S3: Estimated Na excretion in boys (*n* = 128), Table S4: Estimated Na excretion in girls (*n* = 139).
