# Supplementary material for: Placing Salt/Soy Sauce at Dining Tables and Out-Of-Home Behavior Are Related to Urinary Sodium Excretion in Japanese Secondary School Students
Source: Nutrients. 2017 Nov 28;9(12):1290. doi: 10.3390/nu9121290 (PMC5748741; doi:10.3390/nu9121290)
Supplement: Supplementary file 1 [file nutrients-09-01290-s001.pdf]

Supplementary Information

Measured 24 h Na Excretion

Sampling and analysis methods have been described in detail previously [3]. Briefly**, 68** students collected 24 h urine samples twice at an interval of 1 or 2 weeks to derive a formula for estimating 24 h Na excretion. After the first void in the morning on the first day was discarded, the student collected all voids in plastic bottles until the first void next morning. The students recorded the time of each void. The students brought 24 h urines samples in plastic bottles to their schools, and we measured total volume, took 7–8 ml of each sample in a test tube, and refrigerated it until urinalysis. The urinalysis method was the same as that employed for the overnight urine samples. The amount of 24 h sodium (24 h Na; mmol/d) excretion was calculated as follows:

$$\text{Measured Na excretion (mmol/d)} = \text{Na concentration of collected urine (mmol/L)} \times \text{urine volume of collected urine (ml)} \times 24\text{ (h)/collection period (h)}. \quad (1)$$

Measured 24 h Na excretion was calculated using Equation (1) for each 24 h urine collection. An average of the two measurements from each person was used as an individual value. Excluding 2 subjects with incomplete questionnaire, 66 subjects were included for statistical analysis.

Table S1. Measured Na excretion (*n* = 66).

		n	Mean	(Standard deviation)	$p_{for\ trend}$ [1]
Salt placed at dining table	Often	7	166.9	(39.0)	
	Sometimes	8	165.8	(53.5)	0.61
	Rarely	8	138.3	(40.7)	
	Never	42	155.8	(46.7)	
Soy sauce placed at dining table	Often		154.9	(39.0)	
	Sometimes	18	151.0	(45.3)	0.55
	Rarely	15	166.7	(45.0)	
	Never	16	151.8	(55.2)	
Health effect of salt	Disagree	17	141.1	(34.1)	0.18
	Agree	8	158.2	(47.0)	
Salt intake appropriate for health	High	58	147.2	(45.3)	0.46
	Not high	22	160.6	(45.9)	
Japanese intake	Higher than others	44	151.4	(45.9)	0.68
	Not higher	38	162.4	(45.7)	
Discretional seasoning use	Often	28	160.1	(43.3)	
	Sometimes	5	141.2	(50.7)	0.67
	Rarely	13	171.0	(38.8)	
	Never	23	149.3	(48.1)	
Add seasoning on foods	≤2 foods	25	149.8	(41.6)	
	3	13	123.3	(15.5)	
	4	6	155.8	(44.4)	0.72
	5	17	178.4	(51.1)	
	≥6	18	146.3	(44.2)	
Convenience stores	≥2/week	4	148.9	(66.5)	
	1/week	8	149.3	(32.5)	
	<1/week and ≥1/month	20	162.7	(50.6)	0.94
	<1/month	21	154.3	(35.9)	
	Did not buy	13	155.2	(57.6)	
Restaurant	>2/month	5	195.0	(23.5)	
	2/month	12	166.2	(40.4)	
	1/month	14	149.1	(40.5)	0.03
	<1/month and ≥1/6 months	23	162.9	(52.5)	
	<1/6 months	12	125.0	(32.9)	

[1] Adjusted for grade, sex, parent's education, moderate and vigorous physical activity, and body mass index.

Table S2. Effect of interaction terms with sex on estimated Na excretion (*n* = 267).

	p
Salt placed at dining table	0.882
Soy sauce placed at dining table	0.907
Health effect of salt	0.929
Salt intake appropriate for healt	0.917
Japanese intake	0.955
Discretional seasoning use	0.019
Add seasoning on foods	0.525
Covenience stores	0.917
Restaurants	0.893

Other covariates were grade, parent's education, and body mass index.

Table S3. Estimated Na excretion in boys (*n* = 128).

		n	Mean	(Standard deviation)	Model 1 $p_{for\ trend}$ [1]	Model 2 $p_{for\ trend}$ [2]	Model 3 $p_{for\ trend}$ [3]
Salt placed at dining table	Often	23	170.6	(20.5)			
	Sometimes	13	158.7	(29.9)	0.04	0.03	0.02
	Rarely	31	163.4	(22.3)			
	Never	61	158.3	(19.9)			
Soy sauce placed at dining table	Often	50	166.7	(23.3)			
	Sometimes	32	157.3	(21.2)	0.17	0.17	0.17
	Rarely	20	158.6	(25.2)			
	Never	26	160.2	(16.3)			
Health effect of salt	Disagree	37	163.3	(21.6)	0.63	0.84	0.69
	Agree	91	161.2	(22.3)			
Salt intake appropriate for health	High	56	162.3	(20.0)	0.80	0.89	0.98
	Not high	72	161.4	(23.5)			
Japanese intake	Higher than others	82	161.5	(18.6)	0.83	0.57	0.57
	Not higher	46	162.3	(27.2)			
Discretional sauce use	Often	14	156.9	(16.2)			
	Sometimes	36	160.3	(25.4)	0.37	0.38	0.62
	Rarely	36	164.2	(25.7)			
	Never	42	162.6	(16.9)			
Add seasoning on foods	≤2 foods	30	157.6	(23.9)			
	3	23	156.2	(23.4)			
	4	33	165.7	(16.6)	0.14	0.14	0.17
	5	19	167.5	(28.0)			
	≥6	23	162.5	(18.5)			
Convenience stores	≥2/week	19	165.8	(24.6)			
	1/week	26	163.4	(21.8)			
	<1/week and ≥1/month	29	166.4	(23.4)	0.13	0.10	0.09
	<1/month	35	155.7	(21.4)			
	Did not buy	19	159.7	(17.4)			
Restaurant	>2/month	18	169.5	(26.8)			
	2/month	26	166.0	(19.0)			
	1/month	35	159.6	(24.2)	0.07	0.11	0.08
	<1/month and ≥1/6 months	27	156.2	(18.7)			
	<1/6 months	22	160.8	(20.3)			

[1] Model 1 was a crude model, [2] Model 2 was adjusted for grade, and parent's education, and [3] Model 3 was adjusted for grade, parent's education, and body mass index.

Table S4. Estimated Na excretion in girls (*n* = 139).

		n	Mean	(Standard deviation)	Model 1 $p_{for\ trend}$ [1]	Model 2 $p_{for\ trend}$ [2]	Model 3 $p_{for\ trend}$ [3]
Salt placed at dining table	Often	25	160.5	(19.2)			
	Sometimes	10	160.1	(13.1)	0.01	0.04	0.04
	Rarely	25	157.5	(22.2)			
	Never	79	148.9	(21.9)			
Soy sauce placed at dining table	Often	45	157.3	(20.3)			
	Sometimes	24	155.0	(19.4)	0.03	0.09	0.09
	Rarely	29	155.2	(20.9)			
	Never	41	146.6	(23.2)			
Health effect of salt	Disagree	16	156.7	(22.6)	0.50	0.85	0.81
	Agree	123	152.9	(21.3)			
Salt intake appropriate for health	High	48	152.7	(20.5)	0.81	0.63	0.62
	Not high	91	153.6	(22.0)			
Japanese intake	Higher than others	84	151.5	(21.7)	0.21	0.36	0.33
	Not higher	55	156.2	(20.9)			
Discretional sauce use	Often	7	165.1	(9.7)			
	Sometimes	29	156.4	(23.1)	0.00	0.02	0.02
	Rarely	47	158.3	(20.2)			
	Never	56	146.1	(20.7)			
Add seasoning on foods	≤2 foods	30	148.1	(23.0)			
	3	21	146.4	(22.9)			
	4	31	153.4	(17.6)	0.00	0.01	0.01
	5	27	154.8	(20.1)			
	≥6	30	162.0	(21.6)			
Convenience stores	≥2/week	5	164.7	(13.4)			
	1/week	19	155.0	(20.9)			
	<1/week and ≥1/month	37	154.6	(25.4)	0.03	0.27	0.26
	<1/month	42	158.0	(16.6)			
	Did not buy	36	144.1	(21.0)			
Restaurant	>2/month	16	157.2	(18.9)			
	2/month	29	160.3	(24.8)			
	1/month	31	153.4	(18.9)	0.01	0.09	0.10
	<1/month and ≥1/6 months	43	153.1	(22.8)			
	<1/6 months	20	140.6	(13.4)			

[1] Model 1 was a crude model; [2] Model 2 was adjusted for grade, and parent's education; [3] Model 3 was adjusted for grade, parent's education, and body mass index.
